# Supplementary material for: A snapshot of HIV-1 genetic diversity in Dominican Republic in 2024: Predominance of the BCar lineage and first description of a CRF02-AG isolate
Source: PLoS One. 2026 May 8;21(5):e0348313. doi: 10.1371/journal.pone.0348313 (PMC13155586; doi:10.1371/journal.pone.0348313)
Supplement: S1 File — Information data of the phylogenetic trees. S2 Table. Sequences used for subtype B lineage. S3 Table. Complete genome sequences used for subtype CRF02-AG. S4 Table. Accession numbers of the sequences of PR/RT region used for subtype CRF02-AG. S1 Fig. Phylogenetic analysis of the complete genome of HIV-1 for discrimination of the BCar and BPandemic lineage. S2 Fig. Phylogenetic analysis of the PR/RT region of HIV-1 CRF02-AG. (ZIP) [file pone.0348313.s001.zip › S4 Table.docx]

**S4 Table.** Accession numbers of the sequences of PR/RT region used for subtype CRF02-AG.

| AB485635 | GQ200768 | KJ395667 | MT741270 |
| --- | --- | --- | --- |
| AJ286136 | GQ200769 | KJ395674 | MT741271 |
| AY093604 | GQ200770 | KJ395676 | MT741272 |
| AY093605 | GQ200771 | KJ395678 | MT741273 |
| AY093607 | GQ200772 | KJ395679 | MT741274 |
| AY151001 | GQ200773 | KJ395682 | MT741275 |
| AY151002 | GQ200774 | KJ395685 | MT741276 |
| EF042627 | GQ200775 | KJ395686 | MT741277 |
| EF042628 | GQ200776 | KJ395687 | MT741278 |
| EF042629 | GQ200777 | KJ395696 | MT741279 |
| EF042630 | GQ200778 | KJ395698 | MT741280 |
| EF042631 | GQ200779 | KJ395699 | MT741281 |
| EF042632 | GQ200780 | KJ395706 | MT741282 |
| EF042633 | GQ200781 | KJ395707 | MT741283 |
| EF042634 | GQ200782 | KJ395714 | MT741284 |
| EF042635 | GQ200783 | KJ395717 | MT741285 |
| EF042636 | GQ200784 | KJ395721 | MT741286 |
| EF042637 | GQ200785 | KJ936922 | MT741287 |
| EF042638 | GQ200786 | KP688126 | MT741288 |
| EF042639 | GQ200787 | KP688128 | MT741289 |
| EF042640 | GQ200788 | KT737329 | MT741290 |
| EF042641 | GQ200789 | KX357229 | MT741291 |
| EF042642 | GQ200790 | KX466827 | MT741292 |
| EF042643 | GQ200791 | KX466828 | MT741293 |
| EF042644 | GQ200792 | KX466829 | MT741294 |
| EF042645 | GQ200793 | KX466830 | MT741295 |
| EF042646 | GQ200794 | KY581491 | MT741296 |
| EF042647 | GQ200795 | MN116211 | MT741297 |
| EF042648 | GQ200796 | MT741198 | MT741298 |
| EF042649 | GQ200797 | MT741199 | MT741299 |
| EF042650 | GQ200798 | MT741200 | MT741300 |
| EF042651 | GQ200799 | MT741201 | MT741301 |
| EF042652 | GQ200800 | MT741202 | MT741302 |
| EF042653 | GQ200801 | MT741203 | MT741303 |
| EF042654 | GQ200802 | MT741204 | MT741304 |
| EF042655 | GQ200803 | MT741205 | MT741305 |
| EF042656 | GQ200804 | MT741206 | MT741306 |
| EF042657 | GQ200805 | MT741207 | MT741307 |
| EF042658 | GQ200806 | MT741208 | MT741308 |
| EF042659 | GQ200807 | MT741209 | MT741309 |
| EF042660 | GQ200808 | MT741210 | MT741310 |
| EF042661 | GQ200809 | MT741211 | MT741311 |
| EF042662 | GQ200810 | MT741212 | MT741312 |
| EF042663 | GQ200811 | MT741213 | MT741313 |
| EF042664 | GQ200812 | MT741214 | MT741314 |
| EF042665 | GQ200813 | MT741215 | MT741315 |
| EF042666 | GQ200814 | MT741216 | MT741316 |
| EF042667 | JN937025 | MT741217 | MT741317 |
| EF042668 | JN937027 | MT741218 | MT741318 |
| EF042669 | JN937028 | MT741219 | MT741319 |
| EF042670 | JN937031 | MT741220 | MT741320 |
| EF042671 | JN937036 | MT741221 | MT741321 |
| EF042672 | JN937078 | MT741222 | MT741322 |
| EF042673 | JN937086 | MT741223 | MT741323 |
| EF042674 | JN937106 | MT741224 | MT741324 |
| EF042675 | JN937107 | MT741225 | MT741325 |
| EF042676 | JQ514094 | MT741226 | MT741326 |
| EF042677 | JQ514095 | MT741227 | MT741327 |
| EF042678 | JQ514096 | MT741228 | MT741328 |
| EF042679 | JQ514097 | MT741229 | MT741329 |
| EF042680 | JQ514098 | MT741230 | MT741330 |
| EF042681 | JQ514099 | MT741231 | MT741331 |
| EF042682 | KC340123 | MT741232 | MT741332 |
| EF042683 | KC340124 | MT741233 | MT741333 |
| EF042684 | KC340276 | MT741234 | MT741334 |
| EF042685 | KC340377 | MT741235 | MT741335 |
| EF042686 | KC340642 | MT741236 | MT741336 |
| EF042687 | KC340677 | MT741237 | MT741337 |
| EF042688 | KC340684 | MT741238 | MT741338 |
| EF042689 | KF922117 | MT741239 | MT741339 |
| EF042690 | KF922136 | MT741240 | MT741340 |
| EU248477 | KF922145 | MT741241 | MT741341 |
| FJ481696 | KF922148 | MT741242 | MT741342 |
| FJ481699 | KF922167 | MT741243 | MT741343 |
| FJ481711 | KF922174 | MT741244 | MT741344 |
| FJ713217 | KF922192 | MT741245 | MT741345 |
| GQ200744 | KJ395595 | MT741246 | MT741346 |
| GQ200745 | KJ395598 | MT741247 | MT741347 |
| GQ200746 | KJ395600 | MT741248 | MT741348 |
| GQ200747 | KJ395611 | MT741249 | MT741349 |
| GQ200748 | KJ395618 | MT741250 | MT741350 |
| GQ200749 | KJ395622 | MT741251 | MT741351 |
| GQ200750 | KJ395627 | MT741252 | MT741352 |
| GQ200751 | KJ395628 | MT741253 | MT741353 |
| GQ200752 | KJ395631 | MT741254 | MT741354 |
| GQ200753 | KJ395633 | MT741255 | MT741355 |
| GQ200754 | KJ395636 | MT741256 | MT741356 |
| GQ200755 | KJ395638 | MT741257 | MT741357 |
| GQ200756 | KJ395641 | MT741258 | MT741358 |
| GQ200757 | KJ395644 | MT741259 | MT741359 |
| GQ200758 | KJ395647 | MT741260 | MT741360 |
| GQ200759 | KJ395648 | MT741261 | MT741361 |
| GQ200760 | KJ395650 | MT741262 | MW788546 |
| GQ200761 | KJ395651 | MT741263 | MW788547 |
| GQ200762 | KJ395652 | MT741264 | OL624904 |
| GQ200763 | KJ395653 | MT741265 | OR543099 |
| GQ200764 | KJ395659 | MT741266 | OR543111 |
| GQ200765 | KJ395662 | MT741267 | OR543125 |
| GQ200766 | KJ395664 | MT741268 | OR543131 |
| GQ200767 | KJ395666 | MT741269 | OR593741 |
