# Supplementary material for: A snapshot of HIV-1 genetic diversity in Dominican Republic in 2024: Predominance of the BCar lineage and first description of a CRF02-AG isolate
Source: PLoS One. 2026 May 8;21(5):e0348313. doi: 10.1371/journal.pone.0348313 (PMC13155586; doi:10.1371/journal.pone.0348313)
Supplement: S1 File — Information data of the phylogenetic trees. S2 Table. Sequences used for subtype B lineage. S3 Table. Complete genome sequences used for subtype CRF02-AG. S4 Table. Accession numbers of the sequences of PR/RT region used for subtype CRF02-AG. S1 Fig. Phylogenetic analysis of the complete genome of HIV-1 for discrimination of the BCar and BPandemic lineage. S2 Fig. Phylogenetic analysis of the PR/RT region of HIV-1 CRF02-AG. (ZIP) [file pone.0348313.s001.zip › S1 Fig legend.docx]

**S1 Fig. Phylogenetic analysis of the complete genome of HIV-1 for discrimination of the BCar and BPandemic lineage.** Phylogenetic inference was performed by the Maximum Likelihood method, with 1000 bootstrap value, using the substitution model described in Supplemental Table 1. The BCar isolates are shown in blue and the BPandemic ones in red. The accession numbers are shown for each isolate, then the lineage (or subtype different than B) for the reference sequences and the country (Arg: Argentina; Br: Brazil; DemRepCongo: Democratic Republic of Congo; HT: Haiti; TT: Trinidad and Tobago).
