# Supplementary material for: A snapshot of HIV-1 genetic diversity in Dominican Republic in 2024: Predominance of the BCar lineage and first description of a CRF02-AG isolate
Source: PLoS One. 2026 May 8;21(5):e0348313. doi: 10.1371/journal.pone.0348313 (PMC13155586; doi:10.1371/journal.pone.0348313)
Supplement: S1 File — Information data of the phylogenetic trees. S2 Table. Sequences used for subtype B lineage. S3 Table. Complete genome sequences used for subtype CRF02-AG. S4 Table. Accession numbers of the sequences of PR/RT region used for subtype CRF02-AG. S1 Fig. Phylogenetic analysis of the complete genome of HIV-1 for discrimination of the BCar and BPandemic lineage. S2 Fig. Phylogenetic analysis of the PR/RT region of HIV-1 CRF02-AG. (ZIP) [file pone.0348313.s001.zip › S1 Table.docx]

**S1 Table. Information data of the phylogenetic trees**

| **Tree** | **Input data** | **Best-fit model** | **Tree Log-likelihood** |
| --- | --- | --- | --- |
| S1 Fig: Complete genome of HIV-1 subtype B | 29 sequences with 9003 nt sites.  N constant sites: 5025 (55.81%)  N invariant (constant or ambiguous constant) sites: 5025 (55.81)  N parsimony informative sites: 2668  N distinct site patterns: 3158 | GTR+F+R4 | -65427.61 |
| Fig 1A: POL | 33 sequences with 1556 nt sites  N constant sites: 969 (62.27%)  N invariant (constant or ambiguous constant) sites: 969 (62.27%)  N parsimony informative sites: 368  Number of distinct site patterns: 589 | TVM+F+I+R3 | -9881.96 |
| Fig 1B: INT | 34 sequences with 845 nt sites  N constant sites: 563 (66.62%)  N invariant (constant or ambiguous constant) sites: 563 (66.62%)  N parsimony informative sites: 172  N distinct site patterns: 263 | TIM3+F+I+R2 | -4929.38 |
| Fig 1C: VIF | 46 sequences with 632 nt sites  N constant sites: 324 (51.26%)  N invariant (constant or ambiguous constant) sites: 324 (51.26%)  N parsimony informative sites: 218  N distinct site patterns: 330 | GTR+F+R3 | -6387.24 |
| Fig 1D: NEF | 55 sequences with 739 nt sites  N constant sites: 312 (42.21%)  N invariant (constant or ambiguous constant) sites: 312 (42.21%)  N parsimony informative sites: 332  N distinct site patterns: 477 | TPM2u+F+I+G4 | -10384.51 |
| Fig 2: Complete genome of CRF02-AG | 29 sequences with 9003 nt sites  N constant sites: 5025 (55.81%)  N invariant (constant or ambiguous constant) sites: 5025 (55.81%)  N parsimony informative sites: 2668  N distinct site patterns: 3158 | GTR+F+R4 | -65427.61 |
| S2 Fig: PR/RT region of CRF02-AG. Sequences described in S4 Table. | 220 sequences with 969 nt sites  N constant sites: 458 (47.26%)  N invariant (constant or ambiguous constant) sites: 458 (47.26%)  N parsimony informative sites: 427  N distinct site patterns: 596 | GTR+F+I+R5 | -22062.73 |
