# Supplementary material for: A snapshot of HIV-1 genetic diversity in Dominican Republic in 2024: Predominance of the BCar lineage and first description of a CRF02-AG isolate
Source: PLoS One. 2026 May 8;21(5):e0348313. doi: 10.1371/journal.pone.0348313 (PMC13155586; doi:10.1371/journal.pone.0348313)
Supplement: S1 File — Information data of the phylogenetic trees. S2 Table. Sequences used for subtype B lineage. S3 Table. Complete genome sequences used for subtype CRF02-AG. S4 Table. Accession numbers of the sequences of PR/RT region used for subtype CRF02-AG. S1 Fig. Phylogenetic analysis of the complete genome of HIV-1 for discrimination of the BCar and BPandemic lineage. S2 Fig. Phylogenetic analysis of the PR/RT region of HIV-1 CRF02-AG. (ZIP) [file pone.0348313.s001.zip › S2 Table.docx]

**S2 Table .** Sequences used for subtype B lineage.

| **Accession number** | **Country** | **Lineage** |
| --- | --- | --- |
| KU168271 | Democratic Republic of Congo | Subtype D |
| KY658683 | Argentina | BPand |
| AB485638 | USA | BPand |
| AB485641 | Brazil | BPand |
| AY586542 | Cuba | BPand |
| DQ358809 | Brazil | BPand |
| DQ358810 | Brazil | BPand |
| DQ383746 | Argentina | BPand |
| EF637046 | Brazil | BPand |
| FJ195086 | Brazil | BPand |
| KJ704795 | USA | BPand |
| KJ849815 | Brazil | BPand |
| KT427685 | Brazil | BPand |
| KT427754 | Brazil | BPand |
| KT427768 | Brazil | BPand |
| EU839597 | Haiti | BCar |
| EU839598 | Haiti | BCar |
| EU839600 | Haiti | BCar |
| EU839601 | Haiti | BCar |
| EU839602 | Haiti | BCar |
| EU839603 | Haiti | BCar |
| EU839604 | Haiti | BCar |
| EU839606 | Dominican Republic | BCar |
| EU839607 | Trinidad and Tobago | BCar |
| EU839608 | Trinidad and Tobago | BCar |
| EU839609 | Trinidad and Tobago | BCar |
| EU839610 | Trinidad and Tobago | BCar |
| KY658702 | Dominican Republic | BCar |
