# Supplementary material for: A snapshot of HIV-1 genetic diversity in Dominican Republic in 2024: Predominance of the BCar lineage and first description of a CRF02-AG isolate
Source: PLoS One. 2026 May 8;21(5):e0348313. doi: 10.1371/journal.pone.0348313 (PMC13155586; doi:10.1371/journal.pone.0348313)
Supplement: S1 File — Information data of the phylogenetic trees. S2 Table. Sequences used for subtype B lineage. S3 Table. Complete genome sequences used for subtype CRF02-AG. S4 Table. Accession numbers of the sequences of PR/RT region used for subtype CRF02-AG. S1 Fig. Phylogenetic analysis of the complete genome of HIV-1 for discrimination of the BCar and BPandemic lineage. S2 Fig. Phylogenetic analysis of the PR/RT region of HIV-1 CRF02-AG. (ZIP) [file pone.0348313.s001.zip › S3 Table.docx]

**S3 Table.** Complete genome sequences used for subtype CRF02-AG.

| **Accession number** | **Country** | **Subtype** |
| --- | --- | --- |
| AB078005 | USA | B |
| AB231893 | Ghana | G |
| \| AB485633  AB485635  AB485637  AY093604  AY093605  AY093607  AY151001  AY151002  AY371128  AY444811  EU786671  EU884501  FJ670515  JX140645  JX140679  K124792  KY989950  MF157740  MF157742  MK177825  MK177826  MW405338  PV691812  PV691814 \| \| --- \| | Ghana  Djiboui  Liberia  Senegal  West Africa  Germany  Ecuador  Ecuador  Cameroon  USA  Spain  Spain  Spain  Spain  Spain  Germany  Spain  Spain  Spain  Spain  Spain  South Korea  USA  USA | CRF02-AG |
