# Supplementary figures and images for: A snapshot of HIV-1 genetic diversity in Dominican Republic in 2024: Predominance of the BCar lineage and first description of a CRF02-AG isolate
Source: PLoS One. 2026 May 8;21(5):e0348313. doi: 10.1371/journal.pone.0348313 (PMC13155586; doi:10.1371/journal.pone.0348313)

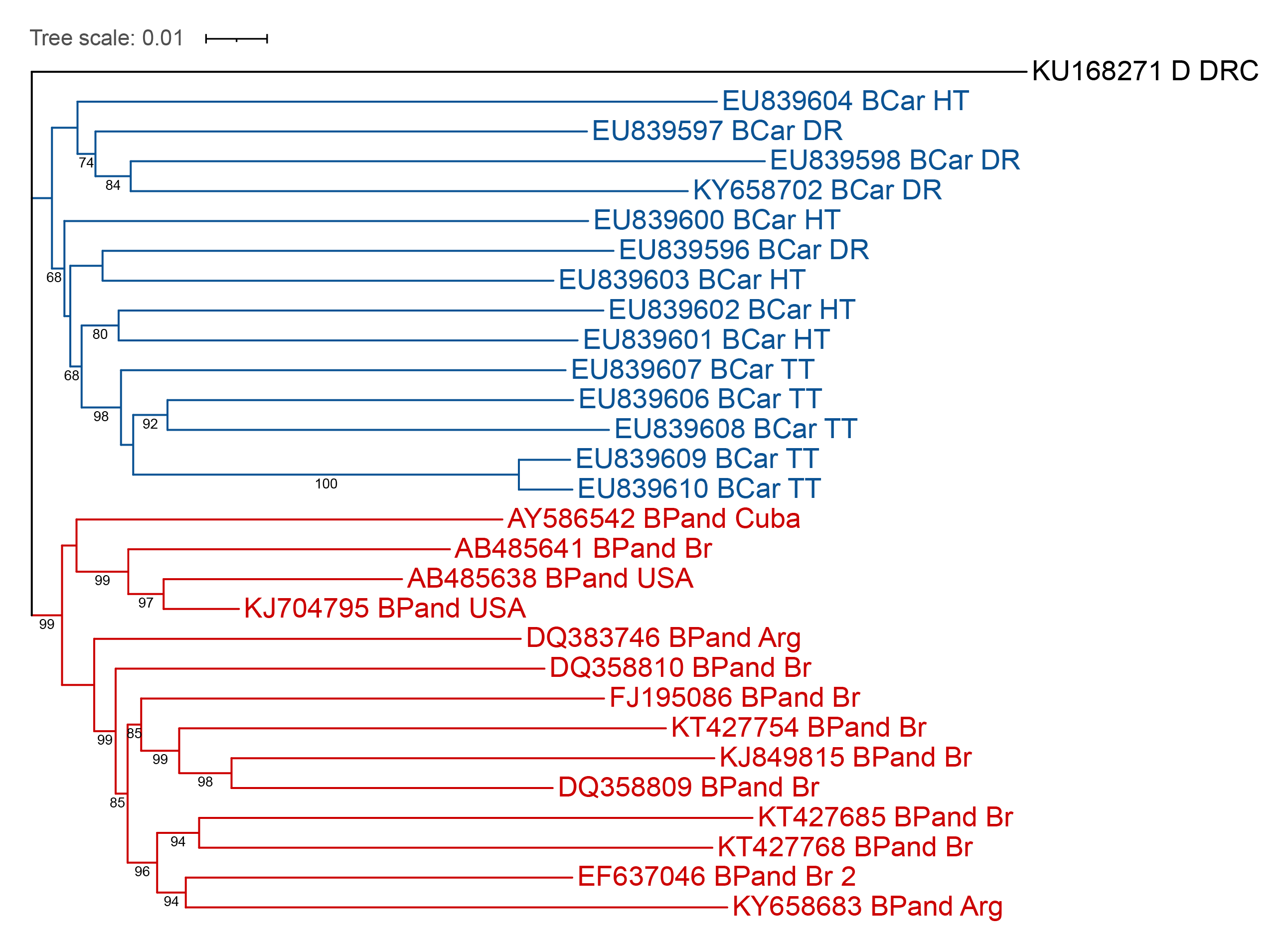

Supplement: S1 File — Information data of the phylogenetic trees. S2 Table. Sequences used for subtype B lineage. S3 Table. Complete genome sequences used for subtype CRF02-AG. S4 Table. Accession numbers of the sequences of PR/RT region used for subtype CRF02-AG. S1 Fig. Phylogenetic analysis of the complete genome of HIV-1 for discrimination of the BCar and BPandemic lineage. S2 Fig. Phylogenetic analysis of the PR/RT region of HIV-1 CRF02-AG. (ZIP) [file pone.0348313.s001.zip › S1 Figure.tif]

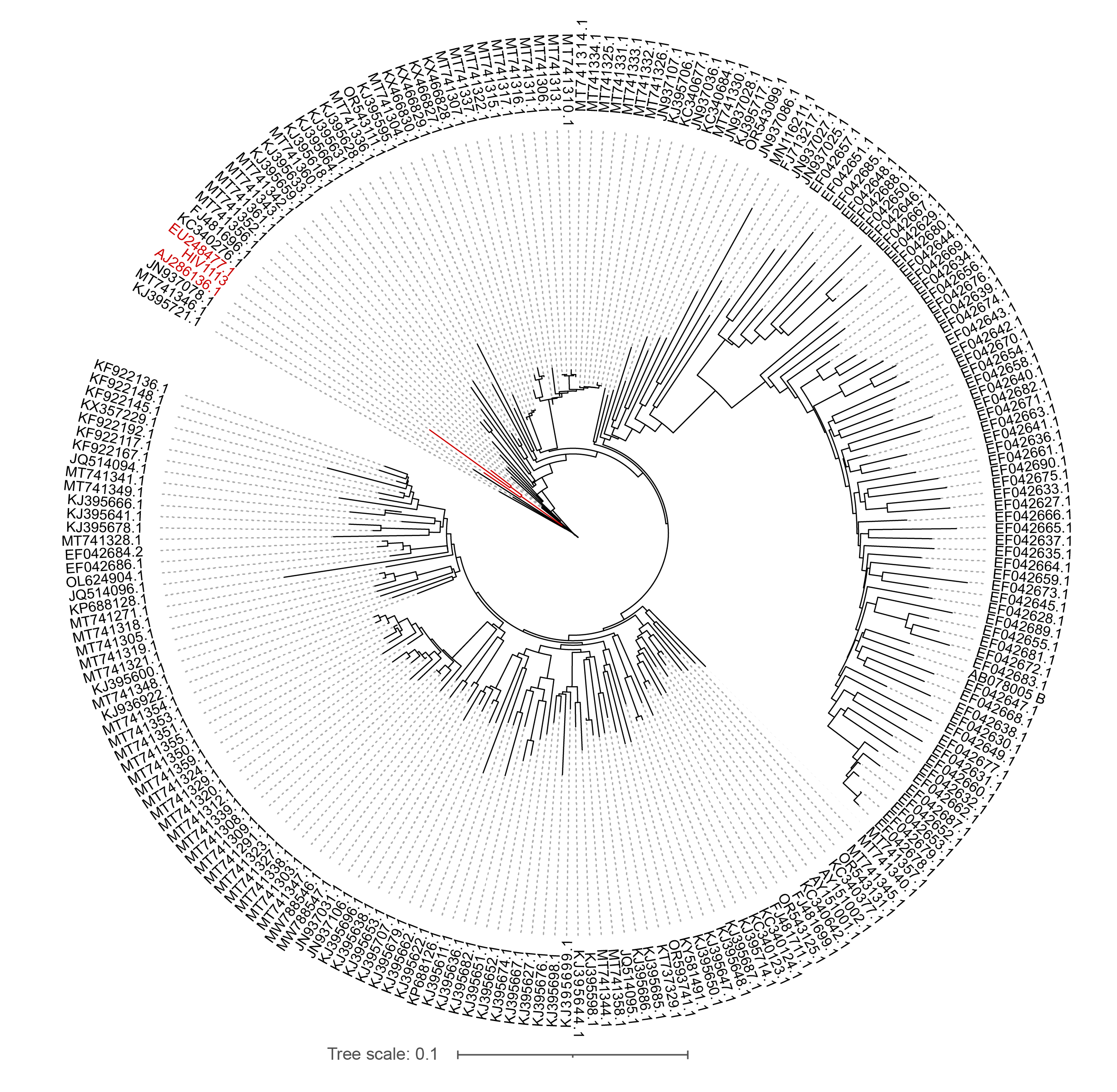

Supplement: S1 File — Information data of the phylogenetic trees. S2 Table. Sequences used for subtype B lineage. S3 Table. Complete genome sequences used for subtype CRF02-AG. S4 Table. Accession numbers of the sequences of PR/RT region used for subtype CRF02-AG. S1 Fig. Phylogenetic analysis of the complete genome of HIV-1 for discrimination of the BCar and BPandemic lineage. S2 Fig. Phylogenetic analysis of the PR/RT region of HIV-1 CRF02-AG. (ZIP) [file pone.0348313.s001.zip › S2 Figure.tif]
