# Supplementary material for: A snapshot of HIV-1 genetic diversity in Dominican Republic in 2024: Predominance of the BCar lineage and first description of a CRF02-AG isolate
Source: PLoS One. 2026 May 8;21(5):e0348313. doi: 10.1371/journal.pone.0348313 (PMC13155586; doi:10.1371/journal.pone.0348313)
Supplement: Attachment — Submitted filename: REVIEWER COMMENTS....pdf [file pone.0348313.s002.pdf]

REVIEWER COMMENTS

REVIEWER : Dr. Christian MANGALA

This manuscript is titled: ". **A snapshot of HIV-1 genetic diversity in Dominican Republic in 2024: predominance of the BCar lineage and first description of a CRF02-AG isolate**", address an important topic from the perspective of the molecular epidemiology of HIV-1 in the Dominican Republic in 2024. However, the manuscript presented by the authors requires minor revisions for improvement.

Abstract

Please specify the type, location, and period of the study. Also specify the techniques used.

Introduction

Line 47: The authors must clearly specify the four groups, that is to say M, O, N and P.

Materials and Methods

- Authors must specify the type and location of studies.
- Please provide the ethics clearance number issued by the ethics committee of the Dominican Republic.
- The “Materials and Methods” section should contain subsections for better clarity.
- Please place the “Ethical Considerations” section after the “Conclusion” section.

Results

The “Results” section should include subsections for clarity.

The figures are not of good quality; authors must submit legible and clear figures.

Conclusion

Authors should include a “Conclusion” section for clarity.

References

Authors must harmonize the list of references, taking into account the standard required by the journal (e.g., number of authors).
